# tRNA-derived small RNAs (tsRNAs): establishing their dominance in the regulation of human cancer

**DOI:** 10.3389/fgene.2024.1466213

**Published:** 2024-11-26

**Authors:** Li Gong, Yajie Hu, Ling Pan, Yufeng Cheng

**Affiliations:** ^1^ Department of Radiation Oncology, Cheeloo College of Medicine, Qilu Hospital of Shandong University, Shandong University, Jinan, Shandong, China; ^2^ Research Center for Basic Medical Sciences, Qilu Hospital of Shandong University, Cheeloo College of Medicine, Shandong University, Jinan, Shandong, China

**Keywords:** tRNA-derived small RNAs, tRNA-derived fragments, tRNA halves, hallmarks, biomarkers, therapeutic targets

## Abstract

The main function of transfer RNAs (tRNAs) is to carry amino acids into the ribosome and synthesize proteins under the guidance of messenger RNAs (mRNAs). In addition to this, it has been observed that tRNAs undergo precise cleavage at specific loci, giving rise to an extensive array of distinct small RNAs, termed tRNA-derived small RNAs (tsRNAs). Existing studies have shown that tsRNAs are widely present across various organisms and comprehensively regulate gene expression, aberrant expression of tsRNAs is inextricably linked to tumorigenesis and development, thus, a systematic understanding of tsRNAs is necessary. This review aims to comprehensively delineate the genesis and expression patterns of tsRNAs, elucidate their diverse functions and emphasize their prospective clinical application as biomarkers and targets for therapy. It is noteworthy that we innovatively address the roles played by tsRNAs in human cancers at the level of the hallmarks of tumorigenesis proposed by Hanahan in anticipation of a broad understanding of tsRNAs and to guide the treatment of tumors.

## 1 Introduction

The researchers recognized that focusing exclusively on the protein-coding exons which constitute less than 2% of the entire genome is insufficient. A significant breakthrough in recent decades has been the exploration of non-coding RNAs (ncRNAs), often referred to as the “dark matter” of the genome, denoting transcripts that do not encode proteins but are now understood to partake in numerous physiological and pathological processes (Alexander et al., 2010; Esteller, 2011). Generally speaking, ncRNAs primarily encompass microRNAs (miRNAs), long non-coding RNAs (lncRNAs), circular RNAs (circRNAs) and others (Slack and Chinnaiyan, 2019). Some relevant reviews have delineated these ncRNAs, each playing an acclaimed role in cellular functions and disease processes, and we will briefly introduce them below (Chen L. et al., 2021; Chen et al., 2022).

Among these, miRNAs have been extensively studied, measuring approximately 21–25 nucleotides in length. miRNAs have the ability to interact with the 3′-untranslated region (3′-UTR) of target mRNA, thereby partnering with Argonaute (AGO) protein to assemble into RNA-induced silence complex (RISC). This culminates in the suppression of target mRNA expression, which plays a very important role in the occurrence and development of the disease (Braun et al., 2012). LncRNAs constitute a bewilderingly complex category of ncRNAs, surpassing 200 nucleotides in length, possess intricate secondary and three-dimensional structures, conferring them with diverse functionalities (Fabbri et al., 2019; Chen Q. et al., 2021; Chen et al., 2022). Interactions between lncRNAs and various nucleic acid molecules (such as DNA, mRNA, other ncRNAs) or proteins (such as histone, RNA-binding protein) have been documented (Wang and Chang, 2011; Wang J. Y. et al., 2020), necessitating further exploration to unravel their mechanisms of action. Additionally, the burgeoning research on circRNAs has extended our comprehension of their biological roles. These circular transcripts are formed by back-splicing, and the downstream splicing site is covalently linked to the upstream splicing site (Dragomir and Calin, 2018; Patop et al., 2019). They are envisioned as super sponges capable of modulating transcription and splicing by binding miRNAs and proteins (Hansen et al., 2013; Liu and Chen, 2022). Further investigations are ongoing to illuminate the full spectrum of functions attributed to circRNAs.

In parallel to the aforementioned ncRNAs, tRNA-derived small RNAs (tsRNAs), have recently garnered heightened attention. They are produced by different ribonucleases that cleave mature or pre-tRNA at specific sites (Lee et al., 2009). Previously dismissed as inconsequential by-products, tsRNAs now represent a class of short RNAs with distinct structures and processing mechanisms (Cole et al., 2009; Garcia-Silva et al., 2010; Peng et al., 2012). They are ubiquitously present across diverse organisms and found to be associated with the development of various diseases, especially cancer (Kumar et al., 2014; Wang J. et al., 2020; Zhang et al., 2021; Lu et al., 2022; Zhang Y. et al., 2022; Xia et al., 2023).

In this comprehensive review, the complex biogenesis and regulatory roles of tsRNAs will be expounded upon, shedding light on their expression patterns, functional significance, and far-reaching implications in tumorigenesis and disease progression. Furthermore, the potential utility of tsRNAs as diagnostic biomarkers and therapeutic targets in oncology will be highlighted, underscoring their emerging role in precision medicine.

## 2 Biogenesis and classification of tsRNAs

**FIGURE 1:**
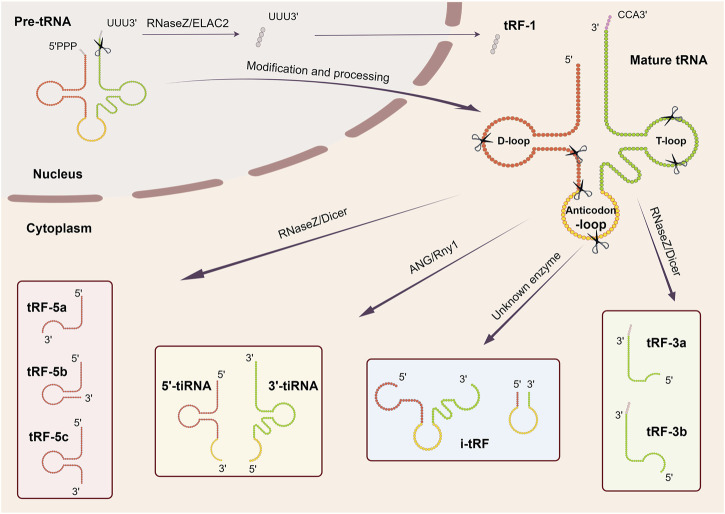
Biogenesis and classification of tsRNAs. tsRNAs are generated from pre-tRNAs and mature tRNAs, which could be further classified into more than six types depending on the different cleavage sites: 5′and 3′tRFs, i-tRFs, tRF-1s, 5′and 3′tRNA halves.

Due to the different conditions and sites at which tRNAs undergo shearing, tsRNAs are categorized primarily into two classes: tRNA-derived fragments (tRFs) and tRNA halves (tiRNAs), which can then be delineated into 5′and 3′tRFs, i-tRFs, tRF-1s, 5′and 3′tRNA halves (Figure 1) (Kim et al., 2020). We will describe each type specifically below. In addition, in view of the nomenclature of tsRNAs has not been uniform across literature, the names of tsRNAs referred to below adhere to those used in the original publications.

### 2.1 5′and 3′tRFs

5′and 3′tRFs are generated by mature tRNAs, and the shear sites are generally in specific regions or after certain nucleotides. 5′tRFs arise from the D-loop or anticodon loop at the 5′end of tRNAs, usually after adenine, a process thought to be mediated by RNaseZs. In contrast, 3′tRFs arise from the 3′end of the tRNAs in the TψC arm, usually between A/U and A/U, and are mediated by a variety of ribonucleases (e.g., RNA polymerase III or angiopoietin) (Lee et al., 2009; Haussecker et al., 2010; Li et al., 2012). Based on their respective lengths, 5′and 3′tRFs can be further classified into different subclasses: tRF-5a (14–16 nt), tRF-5b (22–24 nt), tRF-5c (28–30 nt) and tRF-3a (∼18 nt), tRF-3b (∼22 nt) (Kumar et al., 2014). Identification of ribonucleases contributing to tRFs generation remains an ongoing pursuit. The role of Dicer in this process is still debatable. Cole and his colleagues proved small RNA derived from tRNA^Gln^ is Dicer dependent based on the evidence that when Dicer activity is enhanced, the amount of tRNA^Gln^ cleavage product at ∼20 nt increases while silencing the Dicer expression, the product decreases (Cole et al., 2009). However, contrasting findings revealed that tRF-3 levels remained unaffected in the Dicer knockout 293T cell lines, even exhibiting higher abundance than in their wild-type counterparts (Kuscu et al., 2018). These observations imply a nuanced role for Dicer in the production of tsRNAs, contingent upon distinct cellular environments or tRNA subsets.

### 2.2 i-tRFs

Internal tRNA-derived fragments (i-tRFs), also known as internal tsRNAs or tRF-2s, are derived from the internal regions of mature tRNAs, spanning the anticodon loop and exhibiting variability in length (Kim et al., 2020). This is a newly identified class of tRFs requiring further investigation.

### 2.3 tRF-1s

tRF-1s originate from the 3′tail of pre-tRNA transcripts and are catalyzed by RNaseZ or its homologous endonuclease ELAC2 (Lee et al., 2009; Haussecker et al., 2010). These tsRNAs vary in length and typically start as the end of a mature tRNA and end with an RNA polymerase III transcriptional termination signal (UUUUU, UUCUU, GUCUU, or AUCUU). The length of tRF-1s is dependent on the termination signal position of each pre-tRNA and is widely distributed (Kumar et al., 2014). The first tRF-1s family member to be identified was tRF-1001, which is present in the cytoplasm (Lee et al., 2009). However, how tRF-1s are transported from the nucleus to the cytoplasm and its specific processing process needs further study.

### 2.4 5′and 3′tRNA halves

5′and 3′tRNA halves, generated at the anticodon region of mature tRNAs, are named because they are close to half the length of mature tRNAs. These tRNA halves, commonly known as tiRNAs (Fu et al., 2008), are predominantly produced in response to various stress conditions such as nutrition deficiency, heat shock, hypoxia. tRNA halves can be further divided into two subclasses: 30–35 nt 5′tRNA halves, from the 5′end of tRNA to the anticodon region and 40–50 nt 3′tRNA halves, from the anticodon region to the 3′end of tRNA (Kim et al., 2020; Tao et al., 2020). Angiogenin (ANG), a member of RNase A superfamily, is a widely researched enzyme catalyzing this process in humans (Fu et al., 2008). While in yeast, *Tetrahymena* and Arabidopsis, this process is carried out by the RNase T2 family member Rny1 (Thompson and Parker, 2009; Andersen and Collins, 2012; Megel et al., 2019). Hence, it is apparent that stress-induced tRNA cleavage involves multiple RNases beyond ANG. What’s more, it should be noted that tRNA halves do not only occur under stressful conditions. Studies have also indicated their constitutive expression in various cellular contexts. Peng et al. found that a class of small RNAs were highly enriched in mature sperm from mouse, rat and human and proved that they derived from 5′halves of tRNA^Glu^ and tRNA^Gly^ and named them “mature-sperm-enriched tRNA-derived small RNAs” (mse-tsRNAs). Since these mse-tsRNAs are mainly localized in sperm head, the researchers speculated that they may be involved in the fertilization process (Peng et al., 2012). Later, Honda et al. identified another type of tRNA halves stably expressed in estrogen receptor positive breast cancer and androgen receptor positive prostate cancer cell lines, termed “Sex hormone-dependent TRNA-derived RNAs”. They are generated by angiogenin cleaving aminoacylated mature tRNAs anticodon region (Honda et al., 2015). The above results show that the biogenesis of tRNA halves is a complex and multifaceted process, requiring further exploration and understanding.

## 3 Characteristics of tsRNAs

### 3.1 Location and abundance

The distribution and abundance of diverse tsRNAs in different species, tissues, or organs varied greatly. A comprehensive examination of three types of tsRNAs revealed a predominant prevalence of tRF-5s, followed by tRF-3s, with tRF-1s being the least abundant. In addition, tRF-5s and tRF-3s were present in all species. tRF-1s, however, were found in very low levels in *Drosophila*, *C. elegans*, *Saccharomyces cerevisiae* (Kumar et al., 2014). Next, the researchers separated the small RNA from adult mouse ovary, testis, brain, embryos and embryonic stem cells, observing varying relative levels. Specifically, the content of tRF-5s and tRF-3s exhibited comparable richness in the ovary and embryos, surpassing that of testes and brains. In contrast, tRF-1s displayed lower abundance, with the brain exhibiting the highest levels (Kumar et al., 2014). Subsequent examination of tRF subcellular localization indicated distinct residency patterns, with tRF-5s predominantly found in the nucleus, while tRF-3s and tRF-1s primarily localized to the cytoplasm. The precise subcellular localization of tsRNAs potentially exhibits a strong correlation with both their origin biogenesis and functional roles within the cellular milieu (Kumar et al., 2014). Moreover, through RNA sequencing, Godoy, P. M. systematically characterized the small RNA profiles of 12 human biofluid types. Notably, extracellular tsRNAs displayed heightened levels in bile, urine, seminal plasma, and amniotic fluid, underscoring their potential relevance as biomarkers and providing valuable insights into the biology of extracellular tsRNAs (Godoy et al., 2018).

### 3.2 tRNA modification and tsRNAs biogenesis

The production of tsRNAs is influenced by various factors, including tRNA modification. tRNAs, which are nucleic acids with the highest number of modified components, rely on these modifications for their maturation, stability and function (Motorin and Helm, 2010; Pan, 2018). Hyper-modifications can increase the thermal stability of tRNA, otherwise will lead to tRNAs specific degradation and participation in the formation of tsRNAs (Motorin and Helm, 2010; Schimmel, 2018). This unique feature may improve their stability and applicability as biomarkers.

#### 3.2.1 Methylation

Methylation is the most common type of tRNA modification and is regulated by specific catalytic enzymes, including methyltransferase (writer), demethylase (eraser), and reading proteins (reader) (Dai et al., 2021). The modulation of tRNA results in alterations in its structural stability, playing a contributory role in the genesis of tsRNAs. Notably, post-transcriptional methylation of tRNA serves as a primary upstream mediator influencing the intracellular abundance of tsRNAs. DNA methyltransferase2 (Dnmt2), a prominent member of the DNA methyltransferase family, expressly engaged in the epigenetic modification of cytosine-5 RNA methylation (m5C). Furthermore, within the anticodon loop of tRNA, the methylation site C38 serves as a target for Dnmt2. Dnmt2 shields tRNA substrates from angiogenin-mediated cleavage through the facilitation of methylations at the C38 locus (Schaefer et al., 2010). Subsequent investigations have revealed that Dnmt2 deletion inhibits tRNA m5C and m2G (N2-methylguanosine) levels in mouse sperm on a high-fat diet, thereby remodeling the expression profile of sperm tRNA-derived small RNAs (Zhang et al., 2018). Concurrently, NOP2/Sun RNA methyltransferase 2 (NSun2), an additional methyltransferase responsible for m5C catalysis. Attenuation of NSun2 activity leads to decreased m5C levels, fostering tRNA cleavage by angiogenin and the accumulation of 5′ tRNA-derived small RNA fragments (Blanco et al., 2014). Moreover, the enzyme Bicoid interacting three domain containing RNA methyltransferase (BCDIN3D) participates in the methylation of tRNA^His^. The depletion of BCDIN3D engenders a marked elevation in tRNA^His^ 3′ fragment levels within cellular contexts by unhinging the regulatory mechanism that prevents Dicer-mediated cleavage (Reinsborough et al., 2019). Similarly, downregulation of tRNA methyltransferase two homolog A (TRMT2A) is succeeded by heightened levels of the ribonuclease angiogenin, which cleaves tRNAs at the anticodon region, leading to the accumulation of 5′tRNA-derived stress-induced RNAs (Pereira et al., 2021). Recent insights by García et al. elucidate that the deficiency of tRNA N7-methylguanosine (m7G) transferase methyltransferase-like 1 (METTL1) undermines m7G tRNA methylation and fosters the genesis of 5′tRNA fragments (García-Vílchez et al., 2023). Apart from the aforementioned “writers,” subsequent investigations have identified human AlkB Homolog 1/3 (ALKBH1/ALKBH3), both constituents of the demethylase family, as key players in the demethylation processes concerning 1-methyladenosine (m1A) and 3-methylcytidine (m3C). Elevated levels of their expression render the substrate tRNA more susceptible to cleavage by angiogenin, thereby bolstering the generation of tsRNAs proximal to anticodon domains (Chen et al., 2019; Rashad et al., 2020).

#### 3.2.2 Pseudouridylation

Pseudouridine (Ψ) is a very common type of RNA modification found in all domains of life. Ψ is carried out by a family of evolutionarily conserved pseudouridine synthases (PUSs) (Borchardt et al., 2020). The investigators have substantiated that PUS7, a constituent of the PUSs family, orchestrates the generation of Ψ at the eighth uridine residue (U8) within mTOG--containing tRNA. Depletion of PUS7 precipitates a reduction in Ψ levels within tRNAs, consequently resulting in the dearth of certain 5′tRFs originating from mTOG-containing tRNA species, notably tRNA-Ala, tRNA-Cys, and tRNA-Val (Guzzi et al., 2018).

#### 3.2.3 Queuosine

Queuosine (Q) serves as a hypermodified 7-deaza-guanosine nucleoside situated at position 34 in specific tRNAs, notably at the wobble position with a GUN anticodon (Müller et al., 2019; Patel et al., 2022). This molecular feature, spanning prokaryotic to eukaryotic domains, has been demonstrated by researchers to afford direct safeguarding to the cognate tRNA^His^ and tRNA^Asn^ against ribonucleases, specifically angiogenin cleavage, consequently reshaping the reservoirs of small RNA fragments within human cellular contexts (Wang et al., 2018). As highlighted earlier, the Dnmt2-mediated C38 m5C modification plays a pivotal role in shielding substrate tRNAs from RNA endonuclease-mediated cleavage. Insights suggest that Q potentially augments the protective efficacy of Dnmt2 by bolstering tRNA stability, implying a plausible synergy between these distinct modes of molecular (Müller et al., 2015; Ehrenhofer-Murray, 2017; Tuorto et al., 2018).

In summary, these findings emphasize the critical role of tRNA modification in tsRNA generation; however, more research is needed to fully understand the intricate and interrelated mechanisms that govern tRNA modification and tsRNA generation.

## 4 Function of tsRNAs

**FIGURE 2:**
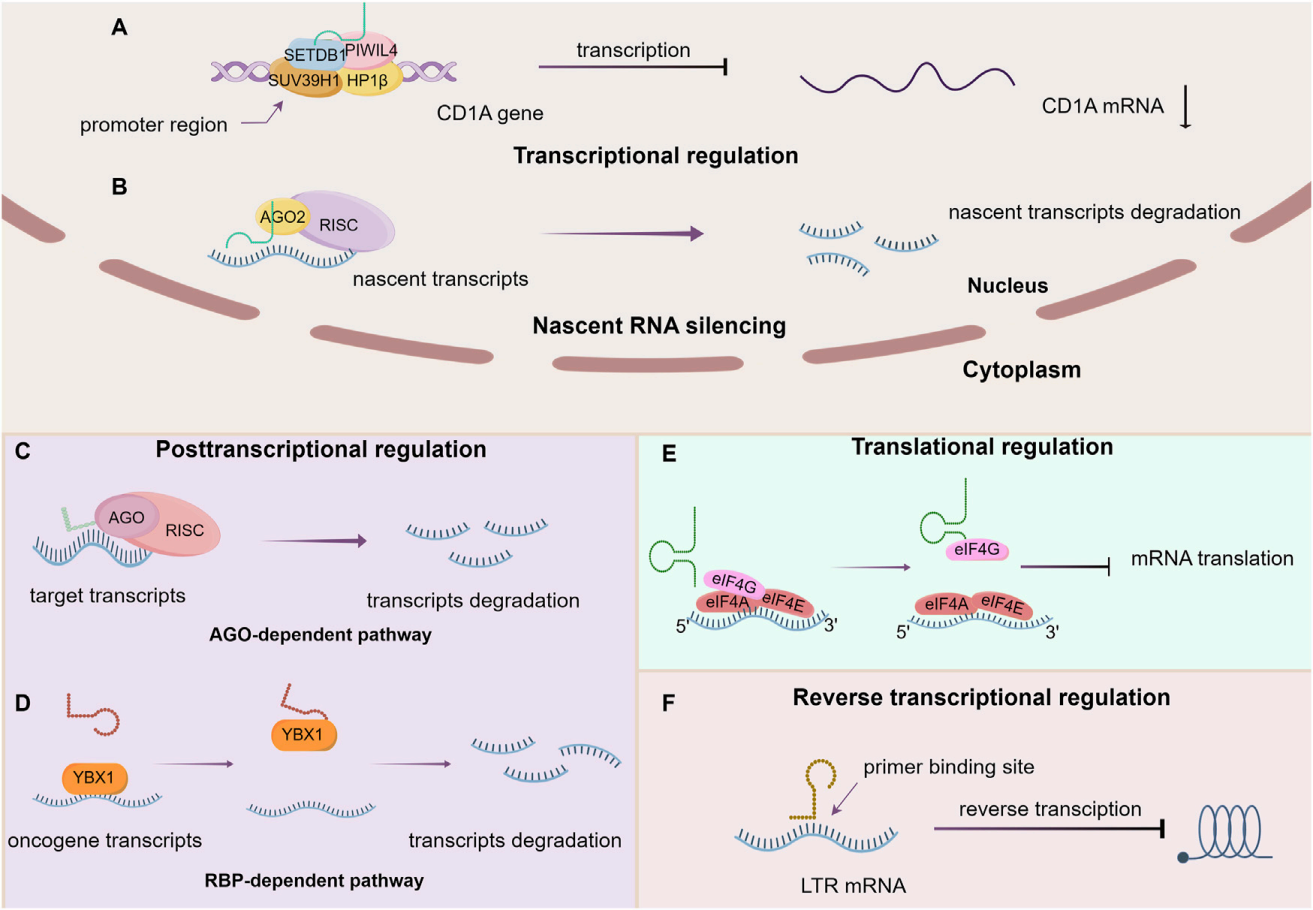
The main function of tsRNAs. **(A)** Transcriptional regulation, **(B)** Nascent RNA silencing, **(C)** AGO-dependent pathway, **(D)** RBP-dependent pathway, **(E)** Translational regulation, **(F)** Reverse transcriptional regulation.

Investigations pertaining to the functionality of tsRNAs remain ongoing. While the definitive impact is yet to be fully elucidated, emerging evidence indicates the involvement of tsRNAs across multiple tiers of gene regulation, encompassing various stages from transcription to post-translation (Figure 2).

### 4.1 Transcriptional regulation

Zhang et al. demonstrated the interaction of a tRNA-Glu–derived piRNA [td-piR (Glu)] with Piwi proteins, which subsequently recruits methyltransferases SET domain-bifurcated histone lysine methyltransferase 1 (SETDB1), suppressor of variegation 3–9 homolog 1 (SUV39H1), and heterochromatin protein 1β to the CD1A promoter region, thereby leading to the methylation of the lysine residue at the ninth position of histone 3 (H3K9). This significantly inhibits the transcription of CD1A in human monocytes (Zhang et al., 2016). Furthermore, specific tsRNAs can also bind to other proteins to modulate histone modification and activate transcription. AS-tDR-007333, which is derived from the D-loop of tRNA^Gly−GCC^, have been shown to directly bind to HSF-binding protein 1 (HSBP1), mediating oncogene Mediator Complex Subunit 29 (MED29) promoter region H3K4me1 and H3K27ac modifications that activate MED29 transcription (Yang et al., 2022).

### 4.2 Nascent RNA silencing

Recent investigations have unveiled a novel mechanism for gene silencing that diverges from established transcriptional gene silencing (TGS) and post-transcriptional gene silencing (PTGS) pathways. This newly identified pathway involves Dicer-dependent tsRNAs, which orchestrate gene downregulation by targeting introns within nascent RNA, a process referred to as nascent RNA silencing (NRS) that primarily occurs in the nucleus. The AGO2 protein serves an essential role in this mechanism. Specifically, tsRNAs guide AGO2-containing silencing complexes to cleave target nascent transcripts, thus hindering their translation into proteins through miRNA-like base pairing. Furthermore, it has been observed that tsRNAs may also influence RNA activation and promoter targeting, as evidenced by the identification of downregulated genes following Dicer and AGO2 depletion. Notably, this nuclear-based mechanism stands apart from PTGS and TGS, as it does not impact the transcriptional state of the gene (Di Fazio et al., 2022).

### 4.3 Post transcriptional regulation

The intricate regulation of post-transcriptional gene expression by tsRNAs exhibits a multifaceted nature, wherein diverse mechanisms contribute to both the enhancement and suppression of gene expression. The latter manifests through three distinct modalities: the AGO-dependent pathway, direct binding mechanisms, and the RNA-binding proteins (RBP)-dependent pathway.

#### 4.3.1 Promote gene expression

The Kim group demonstrated that a specific tsRNA, LeuCAG3′tsRNA, facilitates the binding of at least two ribosomal protein mRNAs (specifically RPS28 and RPS15) through base-pairing interactions at target sites within the mRNA. This binding leads to the enhancement of mRNA translation and acceleration of 18S ribosomal RNA (rRNA) maturation by unfolding the secondary structure of the mRNA through competitive inhibition of target site folding (Kim et al., 2017).

#### 4.3.2 Inhibit gene expression

tsRNAs can exert inhibitory effects over gene expression through a range of mechanisms, which can be categorized as either AGO-dependent or AGO-independent pathways. Regardless of the specific route employed, the overall outcome appears to involve the reduction of mRNA stability, ultimately leading to degradation.

#### 4.3.3 AGO-dependent pathway

Initially, researchers observed that 3′ terminal tsRNAs could engage with the primer binding site (PBS) region of human endogenous retroviral sequences and cleave target transcripts. Despite differing from classical RNA interference (RNAi) due to their independence from Dicer processing, these tsRNAs are capable of associating with and directing AGO2 to downregulate target genes (Li et al., 2012). Subsequently, a specific tRNA-derived fragment, CU1276, originating from human mature B cells, was found to be DICER1-dependent and capable of binding to all four human Argonaute proteins, thus functioning in a miRNA-like manner to post-transcriptionally repress mRNA transcripts (Maute et al., 2013). Additionally, some endogenous 18 nt tRFs generated from the 3′ends of tRNAs (tRF-3) interact with the Argonaute-GW182 complex to assemble into the RISC, thereby targeting and repressing translation of mRNAs in the 3′UTR and promoting their degradation in HEK293T cells (Kuscu et al., 2018). In *Drosophila*, tsRNAs are capable of recognizing and inhibiting specific mRNAs of key components of the general translation machinery, such as ribosomal proteins and initiation elongation factors (IEFs), through interactions with conserved sequences, consequently leading to the inhibition of global mRNA translation. This process is dependent on AGO2 (Luo et al., 2018).

#### 4.3.4 Direct binding

Certain tsRNAs are capable of direct interaction with transcripts, consequently modifying their stability and modulating gene expression. For instance, specific tRF-3 chimeras have been observed to bind to the stem loop of histone mRNAs, thereby influencing mRNA stability and leading to conventional mRNA degradation (Kumar et al., 2014). Additionally, certain tsRNAs have been found to target and cleave endogenous transposable element (TE) mRNAs, serving as components of a genome protection mechanism in plants (Martinez et al., 2017). Moreover, subsequent research has demonstrated that 22 nt tRFs containing a 3′ terminal CCA sequence from mature tRNAs are able to suppress coding-competent long terminal repeat (LTR)-retrotransposons via the PBS target in mice (Schorn et al., 2017).

#### 4.3.5 RBP-dependent pathway

Goodarzi discovered tsRNAs derived from tRNA molecules, including tRNA^Glu^, tRNA^Asp^, tRNA^Gly^, and tRNA^Tyr^, featuring a CU-box motif that interacts with Y-box binding protein 1 (YBX1, stabilize oncogenic transcripts and mediate their enhanced expression). Remarkably, the tsRNAs can competitively displace and destabilize YBX1-bound oncogenic transcripts, leading to their repression and reduced expression (Goodarzi et al., 2015).

### 4.4 Translational regulation

In U2OS cells, 5′-tRNA halves, generated by ANG, have been found to inhibit protein synthesis in a phospho-eIF2α–independent manner (Yamasaki et al., 2009). Subsequent research has confirmed that these tiRNAs achieve translation inhibition by inducing the assembly of stress granules (SGs) (Emara et al., 2010). Furthermore, it has been observed that 5′-P-tiRNA cause a noteworthy reduction in protein synthesis rates in both LKS and MyePro cells (Goncalves et al., 2016). Notably, tsRNAs play a regulatory role in various phases of translation, thereby modulating gene expression.

#### 4.4.1 Initiation

During this phase, translational initiation factors are frequently the focus of targeting mechanisms. Ivanov demonstrated that specific 5′-tiRNAs inhibit protein synthesis by displacing translation initiation factor eIF4G/A from uncapped mRNA or m7G capped mRNA (Ivanov et al., 2011). These 5′tiRNAs contain uniquely stable G-quadruplex structures formed by 5ʹ-terminal oligoguanine motifs (Lyons et al., 2017). Moreover, ribosomes are also susceptible to the impacts of tsRNAs. Over a decade ago, investigators reported that a 26-residue fragment derived from the 5′part of valine tRNA targets the small ribosomal subunit, interfering with peptidyltransferase activity in archaeon and reduces protein synthesis (Gebetsberger et al., 2012). Subsequent studies have shown that under stress conditions in the halophilic archaeon Haloferax volcanii, a valine tRNA derived fragment (Val-tRF) competes with mRNA for ribosome binding and inhibit peptide bond formation, ultimately affecting translation initiation and elongation, resulting in global translation suppression *in vivo* and *in vitro* (Gebetsberger et al., 2017). Additional investigations have demonstrated that tsRNAs target ribosomal proteins and translational initiation or elongation factors, dependent on AGO2 for maintaining energy homeostasis and metabolic adaptation under stress (Luo et al., 2018).

#### 4.4.2 Elongation

Studies by Sobala have revealed that a tRF derived from valine tRNA can impede peptide bond formation and a fraction of cellular 5′tRFs associate with elongating polysomes, indicating their potential to interfere with the elongation process or tRNA charging specificity (Sobala and Hutvagner, 2014). Furthermore, a 19 nt long 5′-tRF obtained from glutaminyl tRNA-Gln19 has been shown to interact with the Multisynthetase complex (MSC, it could deliver charged tRNAs to the ribosome and recycle deacylated tRNAs) in a sequence-independent manner, thereby interfering with or blocking the normal movement of translational substrates and causing a slowdown or stalling of ribosomal complexes during translation. This has led to global translational repression *in vitro* and stimulation of ribosomal and RNA-binding protein translation (Keam et al., 2017). Recently, Li et al. demonstrate that an age-dependent accumulation of Glu-5′tsRNA-CTC, derived from nuclear-encoded tRNA^Glu^ in the mitochondria of glutaminergic neurons. This Glu-5′tsRNA-CTC competitively bind to Leucyl-TRNA Synthetase 2 (LaRs2) with mt-tRNA^Leu^, disrupting the aminoacylation of mt-tRNA^Leu^ and the translation process of mitochondrial protein, further damaging the structure of the mitochondrial cristae and the synthesis of synaptosomal glutamate, thereby accelerating the process of brain aging (Li et al., 2024).

### 4.5 Reverse transcription regulation

According to previous literature, 18 nt tsRNAs have been observed to impede the reverse transcription process of LTR-retrotransposons or endogenous retroviruses (ERV) by perfectly complementing the PBS, including non-autonomous elements (Schorn et al., 2017). Nonetheless, scant research exists on this aspect, emphasizing the necessity for additional exploration and in-depth investigation in this domain.

### 4.6 Post translational regulation

Altering the state of modifications represents a key mechanism by which tsRNAs exert post-translational regulatory functions on pre-existing proteins. Pan, L. discovered that tRF-21 can bind to Ser52 within the Gly-rich domain of the oncogenic RNA-binding protein heterogeneous nuclear ribonucleoprotein L (hnRNP L) in pancreatic cancer. This binding prevents phosphorylation of hnRNP L by AKT2/1 and inhibits the formation of alternative splicing complexes between hnRNP L and dead-box helicase 17 (DDX17). Certain inflammatory cytokines such as leukemia inhibitory factor (LIF) and IL-6 are capable of suppressing the expression of tRF-21, leading to increased production of hnRNP L-DDX17 complex. This, in turn, facilitates selective splicing of Caspase nine and mH2A1 pre-mRNA to generate Caspase 9b and mH2A1.2, ultimately promoting invasion and progression of pancreatic cancer. Consequently, the heightened hnRNP L phosphorylation, stemming from the downregulation of tRF-21, serves as a crucial molecular mechanism driving the onset of malignant biological behaviors in pancreatic cancer (Pan et al., 2021).

## 5 Roles of tsRNAs in cancers

**FIGURE 3:**
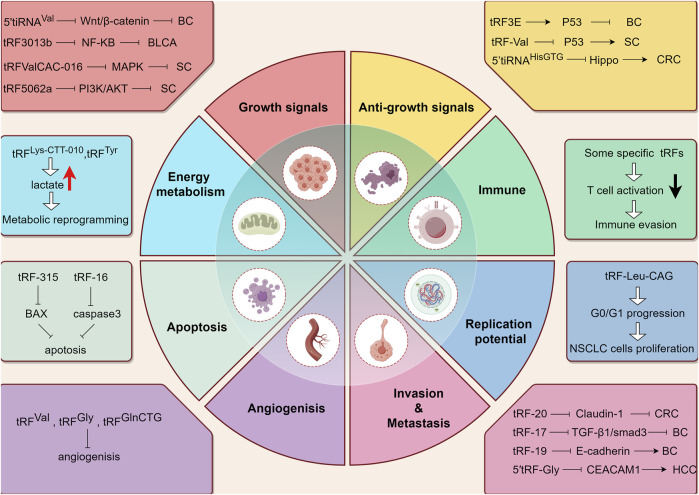
The working mechanism of tsRNAs from the perspective of tumor hallmarks. BC: Breast cancer. BLCA: Bladder Cancer. SC: Stomach Cancer. CRC: colorectal cancer. HCC: hepatocellular cancer. Upward arrow (red) represents upregulation. Downward arrow (black) represents downregulation.

Hanahan elucidates 14 hallmark events intrinsic to tumorigenesis and developmental processes (Hanahan and Weinberg, 2011; Hanahan, 2022), within which we shall delineate the contributory role of tsRNAs (Figure 3).

### 5.1 tsRNAs and growth signal modulation

The ability of tumor cells to perpetually proliferate represents a fundamental hallmark, and its acquisition involves diverse mechanisms, affording them mastery over their fate. This encompasses the generation of an array of growth factor ligands, heightened levels of cell surface receptor proteins, and the activation of downstream signal transduction pathways. As previously elucidated, tsRNAs exert a broad and intricate regulatory influence on gene expression, with diverse tsRNAs exhibiting varied impacts on tumor progression owing to their modulation of distinct endogenous target genes. Notable examples include the inhibition of the Wnt/β-Catenin signaling pathway through the targeting of frizzled class receptor 3 (FZD3) and subsequent reduction in β-Catenin, c-myc, and cyclinD1 levels by 5′-tiRNA^Val^. Consequently, the downregulation of 5′-tiRNAVal in breast cancer demonstrated a positive correlation with malignant phenotypes (Mo et al., 2019). In the same way, tRF3008A, a tRF derived from tRNA^Val^, induces the destabilization of the oncogenic transcript Forkhead Box K1 (FOXK1) in colon cancer cells. Diminished levels of tRF3008A resulted in elevated FOXK1 expression, consequently activating aberrant Wnt/β-catenin signaling and disrupting the expression of C-JUN, c-myc, and cyclinD1, thereby promoting the progression and metastasis of colorectal cancer (Han et al., 2022). Furthermore, the novel 3′-tRF-His-GTG-012, also known as tRF-3013b, exerts a silencing effect on Tumor Protein P63 Regulated 1 Like (TPRG1L) mRNA, the activator of the NF-κB pathway. Decreased tRF-3013b expression correlates with NF-κB pathway activation and dysregulation of cell-cycle proteins such as c-myc, CDK2, hence promoting cell proliferation in gallbladder cancer (Zou et al., 2022). Notably, tRF-Val-CAC-016 markedly inhibits the proliferation of gastric carcinoma cell lines, regulating the classic MAPK signaling pathway by targeting Calcium Voltage-Gated Channel Subunit Alpha1 D (CACNA1d) and suppressing gastric carcinoma proliferation. Reduced expression of tRF-Val-CAC-016 activates CACNA1d-mediated MAPK signaling, thereby enhancing tumor growth (Xu et al., 2022). In instances where the negative feedback mechanism operates normally, it ensures stability within intracellular signaling pathways; however, its impairment can potentiate proliferative signals, thus advancing tumorigenesis. One illustrative case is the Phosphatase and Tensin Homolog (PTEN), known for its negative regulation of the PI3K/AKT signaling pathway, which plays a pivotal role in various physiological functions such as cell proliferation and migration. Zhu et al. discovered downregulation of tRF-5026a in gastric cancer tissues, plasma samples, and cells, accompanied by reduced PTEN expression. This reduction weakens the negative feedback inhibition of the PI3K/AKT pathway, thereby increasing PI3K and AKT expression and ultimately promoting cell proliferation and migration (Zhu L. et al., 2021).

### 5.2 tsRNAs and anti-growth signal modulation

The continued growth of tumor cells can also be attributed to desensitization to growth-inhibiting signals. In the context of breast cancer, it has been observed that tRF3E selectively interacts with RNA-binding protein nucleolin (NCL), displacing p53 mRNA from NCL. This displacement results in enhanced expression of the tumor suppressor gene p53, leading to growth arrest and ultimately cell death. The downregulation of tRF3E attenuates the suppression of tumor growth signal, thereby contributing to the malignant behavior of breast cancer (Falconi et al., 2019). Interestingly, the direct targeting of anti-oncogene by tsRNAs presents positive implications for tumor cell survival and progression, although this is not the result we want to see, but it does exist. For instance, a specific tRNA half, 5′tiRNA-His-GTG directly targets the large tumor suppressor kinase 2 (LATS2) to curb the tumor-suppressive hippo signaling, which inhibits proliferation and promotes apoptosis by regulating Yes-associated protein (YAP) activity. Therefore, in colorectal cancer, heightened expression of 5′tiRNA-His-GTG significantly reduces LATS2 and phospho-YAP (Ser127) levels, thus influencing cancer progression (Tao et al., 2021). Furthermore, the researchers observed that the upregulation of a novel 3′tRNA-derived fragment, tRF-Val, in gastric cancer (GC) tissues is associated with aggressive tumor behavior such as invasion and metastasis. Subsequent research has revealed that tRF-Val targets and mediates Eukaryotic Translation Elongation Factor 1 Alpha 1 (EEF1A1) transport into the nucleus, thereby facilitating the interaction of EEF1A1 with the nuclear-localized MDM2-p53 complex. Eventually, the downstream molecular of p53 is suppressed, rendering tumor cells insensitive to growth-inhibiting signals (Cui et al., 2022).

### 5.3 tsRNAs and apoptosis

Evasion of programmed cell death represents another critical hallmark of tumor cells. Initially, Saikia observed that angiogenin-induced tRNA halves interfaced with Cytochrome c (Cyt c), generating a ribonucleoprotein complex which impeded Cyt c association with apoptotic protease activating factor-1 (Apaf-1). This interaction consequently hindered Cyt c/Apaf-1-mediated activation of the apoptosome, ultimately restraining apoptosis in cells under hyperosmotic stress (Saikia et al., 2014). Subsequently, Chen delineated that m1A demethylated tRNAs were prone to cleavage by ANG in their anticodon regions, leading to the formation of tiRNAs. These tiRNAs facilitated ribosome assembly and established interconnections with Cyt c, rendering cells transfected with such tiRNAs more resistant to stress-induced apoptosis (Chen et al., 2019). Recent studies have increasingly scrutinized the participation of tsRNAs in conferring resistance to chemotherapy drugs by impeding drug-induced cellular apoptosis, thereby fostering the escalation or relapse of diverse malignancies. Specific tsRNAs, exemplified by tRF-315 sourced from tRNA^Lys^, exhibited elevated expression in prostate cancer patients. tRF-315 impeded cisplatin-induced apoptosis by targeting growth arrest and DNA damage inducible alpha (GADD45A), a factor known to promote apoptosis through upregulation of BAX gene expression and downregulation of BCL-2 gene expression (Yang et al., 2021). Additionally, a distinct subset of tRNA-derived fragments, typified by tRF-16-K8J7K1B, assumed pivotal roles in fostering Tamoxifen resistance in hormone receptor-positive (HR^+^) breast cancer. The heightened expression of tRF-16-K8J7K1B potentiated tamoxifen resistance by downregulating the expression of apoptosis-related proteins, including caspase three and poly (ADP-ribose) polymerase, thereby attenuating drug-induced cellular apoptosis (Sun et al., 2023).

### 5.4 tsRNAs and cell replication potential

Normal cells are restricted to a finite and regulated growth-division cycle, in stark contrast to tumor cells which exhibit characteristics akin to the unbounded proliferation typically observed in stem cells. Within mouse embryonic stem cells (mESCs), specific 5′-tsRNAs are capable of delineating stem cell phenotypes by repressing the expression of genes that sustain stemness, thereby facilitating cellular differentiation (Krishna et al., 2019). Furthermore, a study found elevated expression of tRF-Leu-CAG in non-small cell lung cancer (NSCLC) has shown a significant correlation with cancer stage. Inhibiting tRF-Leu-CAG in NSCLC cells resulted in a marked increase in cells present in the interdivision phase compared to control groups, suggesting that tRF-Leu-CAG may induce G0/G1 cell cycle progression and thereby enhance proliferation of NSCLC cells (Shao et al., 2017).

### 5.5 tsRNAs and angiogenesis

We all know that ANG plays a vital role in promoting angiogenesis and facilitating tsRNAs production during hypoxic conditions. However, investigations into the relationship between tsRNAs and angiogenesis have remained infrequent. Deng demonstrated for the first time that tRNA^Val^- and tRNA^Gly^-derived fragments in endothelial cells produced by angiogenin cleavage can inhibit cell proliferation, migration and tube formation and in turn inhibit angiogenesis (Li et al., 2016). Moreover, recent findings by Chen have revealed that the overexpression of tRF^GlnCTG^ restrains the viability and tube-formation capacity of vascular endothelial cells, achieved through the modulation of relative mRNA levels pertaining to vascular endothelial cell markers and pro-angiogenic factors such as Anthrax toxin receptor 1 (Antxr1) (Chen et al., 2023).

### 5.6 tsRNAs and tissue invasion and metastasis

It is widely accepted that the downregulation or loss of expression of critical cell adhesion molecules, such as cadherin, and the upregulation of cell adhesion molecules associated with cellular migration, confer invasiveness and metastatic potential to cancer cells. The process of epithelial-to-mesenchymal transition (EMT) serves as a pivotal environmental signal capable of inducing the infiltrative growth potential in tumor cells. Through the utilization of tRF sequencing and real-time polymerase chain reaction (Real-time PCR) assays, Luan discovered the differential expression of tRF-20-M0NK5Y93 under hypoxic conditions and demonstrated its ability to modulate Claudin-1, an EMT-related molecule, ultimately suppressing metastatic progression in colorectal cancer (CRC) (Luan et al., 2021). Similarly, tRF-17-79MP9PP (tRF-17), originating from the 5′ mature tRNA-Val-AAC and tRNA-Val-CAC, attenuates malignant behaviors in breast cancer cells, including proliferation and migration, by targeting the THBS1/TGF-β1/smad3 axis and EMT-associated genes such as N-cadherin, matrix metalloproteinases3 and matrix metalloproteinases9 (MMP3 and MMP9) (Mo et al., 2021). Given that tsRNAs influence different downstream molecules, their impact on tumor invasion and metastasis varies accordingly. The heightened expression of tRF-19-W4PU732S derived from mature tRNA-Ser-AGA in breast cancer (BC) tissues and cells promotes EMT and cancer stem-cell (CSC) phenotypes, including upregulation of Octamer-binding transcription factor 4 (OCT-4A), SRY-box transcription factor 2 (SOX2) and Vimentin, as well as downregulation of E-cadherin, ultimately facilitating invasion and metastasis of BC cells (Zhang Z. et al., 2022). In mice tumor models wherein tRF-24-V29K9UV3IU is suppressed, the expression of the epithelial cell marker E-cadherin diminishes, while mesenchymal cell markers N-cadherin and vimentin exhibit an opposing trend, thus promoting gastric cancer progression (Wang H. et al., 2022). Moreover, the 5′tRF-Gly intensifies cell growth, migration, and invasion in hepatocellular carcinoma (HCC) by restraining CEA cell adhesion molecule 1 (CEACAM1) (Liu et al., 2022).

### 5.7 tsRNAs and cellular energetics

Metabolic reprogramming stands out as a fundamental characteristic of cancer, demanding that tumor cells adapt their metabolic processes to satisfy the imperative of swift proliferation. The well-recognized Warburg effect typifies this metabolic adaptation, marked by an intensified reliance on glycolysis and substantial lactic acid production even in the presence of oxygen. This metabolic shift significantly contributes to tumor invasion and metastasis (Elia and Haigis, 2021). Notably, Zhu’s investigation unveiled a novel mechanism whereby tsRNAs exert regulatory control over tumor progression by manipulating glucose metabolism. Specifically, tRF^Lys−CTT-010^ exhibited significant upregulation in human triple-negative breast cancer (TNBC) and was found to modulate the expression of glucose-6-phosphatase catalytic subunit (G6PC), a pivotal enzyme in glucose metabolism. Subsequent research elucidated that tRF^Lys−CTT-010^ association with G6PC resulted in the upregulation of cellular lactate production and glycogen consumption, thereby orchestrating a reprogramming of cancer cell glucose metabolism. Augmented lactate levels sustain cancer development by fueling energy production, while also serving as essential substrates for gluconeogenesis, ultimately fostering increased glucose production—a supplementary energy source for tumor growth (Zhu P. et al., 2021). This cascade of events culminates in enhanced cell proliferation, migration, and invasion. An analogous pattern is observed in the context of other tRFs, as evidenced by the significant elevation of tRF^Tyr^ in laryngeal squamous cell carcinoma (LSCC) tissues. Elevated tRF^Tyr^ levels contribute to heightened phosphorylation of lactate dehydrogenase A (LDHA), prompting an accumulation of lactate in LSCC cells and facilitating progression of LSCC tumors (Zhao et al., 2023).

### 5.8 tsRNAs and immune

Upon T cell activation, a surge in CD44 expression and cytokine synthesis, including IL-2, occurs alongside a reduction in CD62L expression. Investigating extracellular vesicles (EVs) emitted by T cells, researchers discovered a significant enrichment of tsRNAs within these vesicles in comparison to the corresponding cellular RNA. Subsequent inquiry revealed that targeting the activation-dependent abundance of 5′tRFs originating from tRNA Leu-TAA and tRNA Leu-TAG via antisense oligonucleotides heightened the proportion of cells exhibiting elevated CD44 expression and IL-2 production while simultaneously displaying diminished CD62L expression. Consequently, the researchers concluded that a specific collection of tsRNAs derived from tRNA 5′ends, 5′-internal regions, and 3′-internal regions without variable loop hairpins, effectively impede T cell activation. T cells selectively release these tsRNAs into exosomes and EVs through their association with multivesicular bodies (MVBs) in a manner regulated by signal, thus swiftly mitigating the presence of tsRNAs and averting their interaction with cytosolic targets known to mediate T cell activation, consequently alleviating their inhibitory impact on immune activation (Chiou et al., 2018). García-Vílchez noted that the absence of METTL1-mediated tRNA methylation instigates tRF production, thereby prompting translational reprogramming of the activated interferon signaling pathway and fostering the polarization of immune cells within the tumor microenvironment (TME) into intratumoral cytotoxic immune cells, such as cytotoxic macrophages and cytotoxic T cells, within Prostate Cancer (PCa) cells. The heightened infiltration of cytotoxic immune cells transmutes the immunosuppressive prostate TME into an intratumoricidal milieu (García-Vílchez et al., 2023).

### 5.9 Others

As a distinctive hallmark newly discovered in tumorigenesis, non-mutant epigenetic reprogramming refers to intrinsic epigenetic regulatory alterations influencing gene expression, primarily originating from the tumor microenvironment and epigenetic regulatory heterogeneity (encompassing DNA methylation, histone modifications, chromatin accessibility, and transcriptional or post-transcriptional RNA modifications). This part has already been covered in the functional section and will not be repeated here. In recent years, polymorphisms in the microbiota are currently a hot topic in biomedical research, and the ecosystems established by certain resident microbiomes have far-reaching implications for health and disease. For tumors, polymorphic variations in the microbiome between individuals in a population may have a profound impact on cancer phenotypes. Reports indicate interactions between certain tsRNAs and the microbiota. Specifically, documented articles feature two tRF variants (tsRNA-000794 and tsRNA-020498) present within human salivary small RNAs, demonstrating inhibitory effects on the proliferation of *Fusobacterium* nucleatum, a significant oral opportunistic pathogen. This inhibitory impact is attributed to the disruption of bacterial protein biosynthesis. Furthermore, it has been observed that F. nucleatum can induce the exosome-mediated release of tsRNA-000794 and tsRNA-020498 by human normal oral keratinocyte cells (He et al., 2018). Certain tRNA halves, particularly tsRNAs originating from non-pathogenic strains of *Escherichia coli*, exhibit potent anti-cancer properties against colorectal cancer, with 50% inhibitory concentration (IC_50_) values at the 10^–8^ M concentratioFin range, compared to the 10^–4^ M range for 5-FU. The cytotoxic effects of tsRNAs derived from the gut microbiota may be achieved through RNAi pathway (Cao et al., 2022).

## 6 Application of tsRNAs in cancer

**FIGURE 4:**
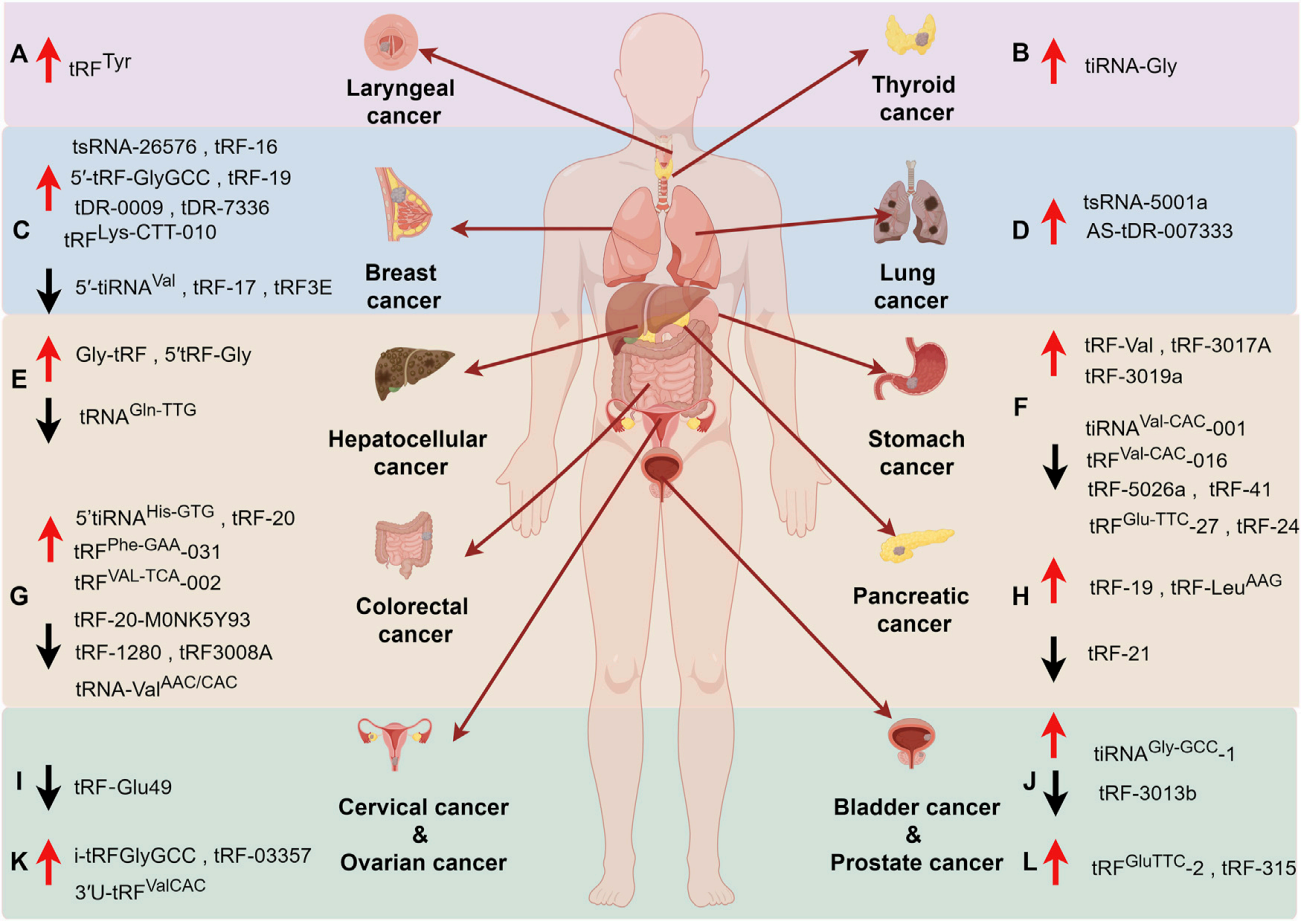
The roles of tsRNAs in cancers. **(A)** Laryngeal cancer, **(B)** Thyroid cancer, **(C)** Breast cancer, **(D)** Lung cancer, **(E)** Hepatocellular cancer, **(F)** Stomach Cancer, **(G)** Colorectal cancer, **(H)** Pancreatic cancer, **(I)** Cervical cancer, **(J)** Bladder Cancer, **(K)** Ovarian cancer, **(L)** Prostate cancer. Upward arrow (red) represents upregulation. Downward arrow (black) represents downregulation.

Combined with the previous section, we already know that tsRNAs are abnormally expressed in a variety of tumors and play very important roles in many pathways involved in tumorigenesis (Figure 4). These properties suggest that tsRNAs have a wide range of applications, such as acting as prospective biomarkers for tumor diagnosis and evaluation of prognosis, or targeting of a specific tsRNA molecule for the treatment of a disease.

### 6.1 Potential diagnosis and prognosis biomarkers

The domain of biomarkers utilized in cancer detection is a well-explored area with established markers like carcinoembryonic antigen (CEA), Carbohydrate antigen 125 (CA125) and Carbohydrate antigen 199 (CA199) widely employed in colon cancer, pancreatic cancer, and various other malignancies. Nevertheless, many prevailing biomarkers exhibit inherent limitations, notably pertaining to suboptimal sensitivity and specificity, thereby constraining their utility in early tumor diagnosis (Duffy et al., 2010). Consequently, there exists a pressing need to identify novel tumor markers that can augment the efficacy of tumor detection methodologies. Based on what we mentioned earlier, it is reasonable to assume that tsRNAs may be very promising tumor markers. Up to now, many articles have reported striking changes in the expression levels of tsRNAs either intracellularly or extracellularly in a wide range of tumors, thus becoming the basis for tumor diagnosis (Table 1).

**TABLE 1:** tsRNAs can be used as biomarkers for tumor diagnosis and prognosis.

Sample type	tsRNA type	Cancer type	Expression characteristics	Reference
Exosome	tRF-Leu-TAA-005 tRF-Asn-GTT-010 tRF-Ala-AGC-036 tRF-Lys-CTT-049 tRF-Trp-CCA-057	NSCLC	All downregulated	Zheng et al. (2022)
	tRNA-ValTAC-3 tRNA-GlyTCC-5 tRNA-ValAAC-5 tRNA-GluCTC-5	LIHC	All upregulated	Zhu et al. (2019)
	tRF-3030b tRF-5008b tRF-3022b	CRC	All upregulated	Lu et al. (2022)
	tRNA-GlyGCC-5	ESCC	Upregulated	Li et al. (2022)
Plasma	tRF-55:74-chrM.Phe-GAA tRF-56:75-Ala-CGC-1-M4	ESCC	All upregulated	Huang et al. (2023)
	tRF-1:29-Pro-AGG-1-M6 tRF-55:76-Tyr-GTA-1-M2	LUAD	Downregulated Upregulated	You et al. (2022)
	tRF-Arg-CCT-017 tRF-Gly-CCC-001 tiRNA-Phe-GAA-003	BC	All upregulated	Wang et al. (2021)
	tRF-Glu-CTC-003 tRF-Gly-CCC-007 tRF-Gly-CCC-008 tRF-Leu-CAA-003 tRF-Ser-TGA-001 tRF-Ser-TGA-002	Early-stage BC	All downregulated	Wang et al. (2020a)
	tRF-27-87R8WP9N1E5	GC	Upregulated	Yu et al. (2022b)
	5'-tRF-GlyGCC	CRC	Upregulated	Wu et al. (2021)
Serum	tRF-23-Q99P9P9NDD	GC	All upregulated	Zhang et al. (2022a)
	tRF-Gln-TTG-006	LIHC	Upregulated	Zhan et al. (2022)
	tRF-Pro-AGG-004 tRF-Leu-CAG-002	PDAC	All upregulated	Jin et al. (2021)
Tissue	tRF-17-79MP9PP	BC	All downregulated	Mo et al. (2021)
	5'-tiRNA-Val 5'-tiRNA-ProTGG i-tRF-GlyGCC	CRC	Upregulated Upregulated Downregulated	Li et al. (2019) Tsiakanikas et al. (2022) Christodoulou et al. (2023)
	5'-tRF-LysCTT	BLCA	Upregulated	Papadimitriou et al. (2020)
Cell	i-tRF-GlyCCC	CLL	Downregulated	Katsaraki et al. (2019)
Extracellular vesicles	tRF-1:30-Lys-CTT-1-M2	Hypopharyngeal Cancer	Upregulated	Xi et al. (2021)

NSCLC: Non-small cell lung cancer. LIHC: Liver hepatocellular carcinoma. CRC: Colorectal cancer. ESCC: Esophageal squamous carcinoma. LUAD: lung adenocarcinoma. BC: breast cancer. GC: Gastric cancer. PDAC: Pancreatic ductal adenocarcinoma. BLCA: Bladder cancer. CLL: Chronic lymphocytic leukemia.

#### 6.1.1 Intracellular tsRNAs

Presently, investigations pertaining to the quantification and assessment of tsRNAs within neoplastic cells predominantly focus on hematologic malignancies. Within this domain, Veneziano and colleagues have identified a substantial decline in the abundance of a tRF-5 derivative from tRNA^His^ during an extensive exploration of tsRNAs expression patterns in Chronic lymphocytic leukemia (CLL). Subsequent research has unveiled notable alterations in the profiles of nearly 1,000 mature tsRNAs in CLL, underscoring the potential of mature tsRNAs as discerning indicators for diagnosis and prognosis of CLL (Veneziano et al., 2019). Moreover, heightened expressions of specific i-tRFs like i-tRF-Gly^GCC^, i-tRF-Phe^GAA^, alongside other tsRNAs variants such as tRF-Leu^AAG/TAG^, have been systematically linked to adverse prognostic outcomes in CLL patient cohorts (Karousi et al., 2019; Karousi et al., 2020; Katsaraki et al., 2021). Additionally, Balatti found that ts-46 and ts-47 are downregulated in CLL and lung cancer (Balatti et al., 2017). Li, S elucidated the emergence of a novel 5′-tiRNA species derived from mature tRNA-Val (5′-tiRNA-Val) exhibiting notable upregulation in colorectal cancer (CRC) individuals with metastasis, thereby establishing a framework wherein tsRNAs enable prognostic assessments in cancer populations. This pioneering work has since catalyzed a continuous influx of scholarly investigations in this domain (Li et al., 2019). Furthermore, Papadimitriou’s seminal discovery of 5′-tRF-LysCTT as a prognostic marker in Bladder cancer (BlCa) highlighted its substantial dysregulation in the disease context, associating heightened levels of 5′-tRF-LysCTT with augmented tumor migration, invasion, and unfavorable treatment outcomes (Papadimitriou et al., 2020). Afterwards, Tsiakanikas brought to light the pivotal role played by elevated levels of 5′-tiRNA-Pro^TGG^ in colorectal cancer (CRC), significantly influencing patient survival and short-term recurrence (Tsiakanikas et al., 2022). Following this, the identification of i-tRF-GlyGCC, in CRC unveiled its autonomous prognostic utility irrespective of conventional clinicopathological factors. Strikingly, i-tRF-GlyGCC demonstrated substantial under-expression in CRC cases, with elevated expression correlating with shorter disease-free survival (DFS) and overall survival (OS) time intervals (Christodoulou et al., 2023).

#### 6.1.2 extracellular tsRNAs (cell-free RNA)

A novel technique known as liquid biopsy has emerged as a method for quantifying molecular biomarkers within biological fluids such as blood, saliva, and urine, circumventing the need for cancer tissue samples (Poulet et al., 2019; Chen Q. et al., 2021; Umu et al., 2022). This approach represents a groundbreaking advancement in cancer diagnosis and prognosis prediction due to its simplicity and non-invasive characteristics (Yu D. et al., 2022). Tumor-derived DNA, RNA, and proteins, including the tsRNAs we are talking about today, could serve as potential biomarkers implicated in tumorigenesis. We have already described that the aberrant expression of tsRNAs is inseparable from tumorigenesis and development. As circulating cell-free RNAs (cfRNAs), tsRNAs may occur in many forms: as free molecules alone, encapsulated in membrane vesicles like exosomes, or forming complexes with proteins and lipids. The distinctive repertoire and quantities of specific tsRNAs discernible in various bodily fluids are contingent upon a myriad of factors, inclusive of cancer type and stage (Kolenda et al., 2020).

#### 6.1.3 Exosome or Extracellular vesicles

Extracellular vesicles containing diverse tsRNAs have been identified across various bodily fluids, including blood, saliva, urine, etc., exhibiting crucial biological functions. Zhu, L conducted a comparative analysis of tsRNAs profiles within plasma exosomes from individuals with liver cancer and healthy donors, revealing differential expressions of four tsRNAs species (tRNA-ValTAC-3, tRNA-GlyTCC-5, tRNA-ValAAC-5, and tRNA-GluCTC-5) in the former cohort. Significantly heightened levels of these tsRNAs in liver cancer patient exosomes underscore their potential diagnostic utility for liver cancer and envisage their promise as liquid biopsy markers (Zhu et al., 2019). In a complementary investigation, Lu, S and colleagues corroborated elevated levels of three distinct tsRNAs (tRF-3022b, tRF-3030b, and tRF-5008b) within both colonic tissues and plasma exosomes of individuals with colon cancer relative to controls, hinting at their putative involvement in tumorigenesis. Notably, tRF-3022b was evidenced to impede M2 macrophage polarization by binding certain cytokines, thus influencing tumor progression dynamics (Lu et al., 2022). Li, K et al. elucidated the presence of two previously unexplored small RNAs, namely, tRNA-GlyGCC-5 and sRESE, within salivary exosomes, denoted as salivary exosomal small non-coding RNAs (sesncRNAs). They formulated a dual sesncRNA signature serving as a non-invasive diagnostic and prognostic tool for esophageal squamous cell carcinoma (ESCC), offering insights into patient survival and the efficacy of adjunctive therapeutic interventions for ESCC. Markedly escalated levels of this dual sesncRNA signature were observable in individuals afflicted with cancer, while transfection with specific antisense RNA effectively impeded various malignant cellular activities *in vitro* with high sensitivity and specificity (Li et al., 2022). All in all, these findings underscore the potential of tsRNAs as a straightforward and non-invasive modality for cancer screening.

#### 6.1.4 Plasma

Wang et al. conducted a comprehensive examination of specific tsRNAs originating from the 5′ends of tRNAs within plasma specimens from early-stage breast cancer (EBC) and revealed a panel of downregulated tRFs, namely, tRF-Glu-CTC-003, tRF-Gly-CCC-007, tRF-Gly-CCC-008, tRF-Leu-CAA-003, tRF-Ser-TGA-001, and tRF-Ser-TGA-002. At the same time, they highlighted a correlation between reduced levels of tRF-Glu-CTC-003 and adverse outcomes in HER2^+^ EBC individuals, indicative of poor disease-free survival and overall survival (Wang J. et al., 2020). Subsequently, in a follow-up study, a distinct cohort analysis involving 120 subjects with Breast cancer (BC) and 112 healthy controls unveiled three novel tsRNAs, namely, tRF-Arg-CCT-017, tRF-Gly-CCC-001, and tiRNA-Phe-GAA-003, as promising diagnostic markers. Of particular interest, elevated expressions of tRF-Arg-CCT-017 and tiRNA-Phe-GAA-003 were associated with favorable prognostic implications, strengthening their roles as prognostic indicators (Wang et al., 2021). Meanwhile, investigations on gastric cancer cohorts indicated a significant elevation in tRF-27–87R8WP9N1E5 (tRF-27) levels in patients compared to controls, and more interestingly, tRF-27 levels decreased and tended to be consistent with levels of healthy individuals in postoperative gastric cancer patients compared to preoperative patients (Yu X. et al., 2022).

Moreover, an analysis focusing on lung adenocarcinoma (LUAD) revealed pivotal tsRNAs, including tRF-16-L85J3KE, tRF-21-RK9P4P9L0, and tRF-16-PSQP4PE, with distinct expression patterns between patient plasma samples and normal controls. In addition, the prognostic significance of tRF-21-RK9P4P9L0 was underscored, as its inhibition demonstrated notable impacts on cancer cell proliferation, migration, and invasion capabilities (Wang J. et al., 2022). In the realm of colorectal cancer (CRC), where CEA and CA199 traditionally serve as primary tumor biomarkers, the emergence of 5′-tRF-GlyGCC as a markedly elevated entity in CRC patient plasma warrants attention. Importantly, the positive correlation observed between 5′-tRF-GlyGCC levels and those of CEA and CA199 further accentuates its diagnostic value, surpassing the individual diagnostic capacities of CEA and CA199. The collective integration of 5′-tRF-GlyGCC with conventional markers such as CEA and CA199 promises enhanced diagnostic efficacy within the CRC diagnostic landscape (Wu et al., 2021).

#### 6.1.5 Serum

Peng, E. Y conducted a pioneering investigation into circulating tRNA-derived small non-coding RNAs (tsncRNAs), unearthing distinct profiles of four tsncRNAs (ts1-4) in the serum of ovarian cancer patients compared to healthy individuals. These tsncRNAs demonstrate a capacity for precise prediction of aberrant cellular proliferation, thereby positioning them as promising candidate biomarkers for the assessment of ovarian cancer (Peng et al., 2018). In pancreatic cancer (PC), a newly identified serum signature comprising tRF-Pro-AGG-004 and tRF-Leu-CAG-002 has been validated as valuable diagnostic biomarker, exhibiting efficacy even in early-stage disease detection. Moreover, this two-tsRNAs signature shows promise in predicting postoperative survival time for PC patients (Jin et al., 2021). Particularly, the heightened expression of tRF-Gln-TTG-006 in serum samples from Hepatocellular carcinoma (HCC) patients compared to healthy controls underscores its potential as a sensitive and specific discriminator of HCC (Zhan et al., 2022). In gastric cancer (GC), the elevated levels of tRF-23-Q99P9P9NDD exhibit a significant association with TNM stage and instances of nerve/vascular infiltration. Furthermore, the upregulation of tRF-23-Q99P9P9NDD correlates with inferior survival outcomes than those with low expression of this biomarker (Zhang Y. et al., 2022).

### 6.2 Therapeutic targets

The uncontrolled tsRNAs found in a variety of cancers can not only be used as biomarkers for the diagnosis and evaluation of prognosis of tumorigenesis, it is important to explore the possibility of tsRNAs as tumor therapeutic targets. Other than that, drug resistance is one of the main reasons for cancer treatment failure, and suppressing or slowing down the emergence of drug resistance will open up new horizons for cancer treatment. Two tsRNAs tDR-0009 and tDR-7336 derived from tRNA^Gly−GCC^ are significantly upregulated in hypoxia-treated Triple-negative breast cancer (TNBC) cell lines. Further studies have shown that these tsRNAs are involved in doxorubicin resistance in TNBC by regulating the activity of phosphorylation of STAT3 (Cui et al., 2019) Additionally, upregulation of tRF-16-K8J7K1B facilitates tamoxifen resistance. Extracellular tRF-16-K8J7K1B can be packaged into exosomes and propagated tamoxifen resistance to recipient cells by targeting apoptosis-related proteins such as caspases three and poly (ADP-ribose) polymerases. The authors also demonstrated that inhibition of the exosome tRF-16-K8J7K1B is able to increase the sensitivity of breast cancer cells to tamoxifen *in vivo*. Therefore, the exosome tRF-16-K8J7K1B may be a powerful therapeutic target to combat tamoxifen resistance in HR^+^ breast cancer (Sun et al., 2023). Not only in breast cancer, tRF-315 derived from tRNA^Lys^, are highly expressed in prostate cancer patients and prostate cancer cells lines. tRF-315 can mediate cisplatin resistance through preventing or alleviating cisplatin-induced apoptosis and mitochondrial dysfunction in cancer cells. At the same time, the authors proved transfection of tRF-315 inhibitor increased the expression of apoptotic related proteins. Therefore, tRF-315 is expected to be a promising therapeutic target in the future (Yang et al., 2021). Whether these new biomarkers can be applied in the clinic and benefit patients will be the focus of future research.

## 7 Conclusion and future perspectives

Non-coding RNAs (ncRNAs) are now a hot topic of research, referring to transcripts that cannot be translated to produce proteins, including microRNAs (miRNAs), long non-coding RNAs (lncRNAs), circular RNAs (circRNAs) and so on. tRNA-derived small RNAs (tsRNAs) represent a novel class of sncRNAs that are produced by the precise cleavage of mature or precursor tRNAs at specific sites by different ribonucleases. Overall, tsRNAs can be divided into six categories based on different sites cleaved by different shear enzymes: 5′and 3′tRFs, i-tRFs, 5′and 3′tRNA halves and tRF-1s. The first five classes are all derived from mature tRNAs, which are produced from the 5′or 3′ends of tRNAs, internally, and half of the tRNAs, respectively. The last class arises from 3′tailer of pre-tRNAs. The expression of different tsRNAs in different tissue or cells varies greatly, and their production is also affected by many factors, such as the modification status of tRNA, especially the methylation level. It seems that different tsRNAs assume important but different functions, but they all exert macro-regulation at various levels of gene expression. Among them, the post-transcriptional regulation of tsRNAs is worth mentioning, the association of tsRNAs to target mRNAs could be either direct (through sequence complementarity) or indirect (through their association with certain RBP). And due to the different downstream molecules acted, the expression of the targets is enhanced or inhibited. As a result, tsRNAs have different effects on disease progression such as tumors.

According to the hallmarks theory of tumors proposed by Hanahan, malignant tumors have 14 significant features in their occurrence and development. We are pleasantly surprised to find that the mechanisms of action of tsRNAs on tumors seem to be attributed to these hallmarks. Our full understanding of the function of tsRNAs also points the way for their clinical application. First of all, tsRNAs in body fluids may serve as potential diagnosis and prognosis biomarkers. Liquid biopsy is convenient and non-invasive, which is conducive to early diagnosis, individualized treatment and prognosis monitoring. However, common tumor markers such as CEA, CA125, and CA199 lack the sensitivity and specificity for diagnosis. We discovered that tsRNAs are dysregulated in a variety of solid tumors or hematologic tumors and are inextricably linked to patient survival. On the other hand, the multiple effects of tsRNAs on tumors make them promising therapeutic targets. But there is still a long way to go before these markers can be applied to the clinic, one of the main challenges is the small molecular weight and low abundance, which makes them difficult to detect, while also considering the uniqueness and applicability of tsRNAs in different tumors. Thus, more researches are needed to verify whether they can be implemented.
